# Feasibility of plant-expression system for production of recombinant anti-human IgE: An alternative production platform for therapeutic monoclonal antibodies

**DOI:** 10.3389/fpls.2022.1012583

**Published:** 2022-12-02

**Authors:** Oranicha Hanittinan, Kaewta Rattanapisit, Ashwini Malla, Kittipan Tharakhet, Chutitorn Ketloy, Eakachai Prompetchara, Waranyoo Phoolcharoen

**Affiliations:** ^1^ Center of Excellence in Plant-produced Pharmaceuticals, Chulalongkorn University, Bangkok, Thailand; ^2^ Department of Pharmacognosy and Pharmaceutical Botany, Faculty of Pharmaceutical Sciences, Chulalongkorn University, Bangkok, Thailand; ^3^ Baiya Phytopharm Co., Ltd., Bangkok, Thailand; ^4^ Department of Laboratory Medicine, Faculty of Medicine, Chulalongkorn University, Bangkok, Thailand; ^5^ Center of Excellence in Vaccine Research and Development (Chula VRC), Faculty of Medicine, Chulalongkorn University, Bangkok, Thailand

**Keywords:** asthma, immunoglobulin E, monoclonal antibody, respiratory ailments, recombinant proteins, transient expression

## Abstract

Omalizumab, the anti-immunoglobulin IgE antibody is the only approved and available monoclonal antibody as an auxiliary medicament for the severe respiratory allergic reactions. It forms small size immune complexes by binding to free IgE, thereby inhibiting the interaction of IgE with its receptors. Additionally, the anti-IgE can also differently shape the airflow by impeding the stimulation of IgE receptors present on structural cells in the respiratory tract. The present study aimed to use plants as an expression system for anti-human IgE antibody production, using *Nicotiana benthamiana* as hosts. Recombinant *Agrobacterium tumefaciens* containing heavy chain (HC) and light chain (LC) domains of anti-human IgE were co-transformed in *N. benthamiana*. The assembling of the antibody and its expression was detected by SDS-PAGE and Western blot analysis. The functional ability of the anti-IgE antibody was determined *via* its binding capacity with target IgE by ELISA and the inhibition of basophil activation. The anti-human IgE mAb generated in plants was shown to be effective in binding to its target IgE and inhibit the IgE-crosslink in RS-ATL8 reporter cells. Although, antibody yield and purification process have to be further optimized, this study demonstrates the use of plant expression system as a promising platform for the production of Omalizumab which showed a comparable *in vitro* function to that of commercial Omalizumab (Xolair) in the inhibition of basophil activation.

## Graphical Abstract

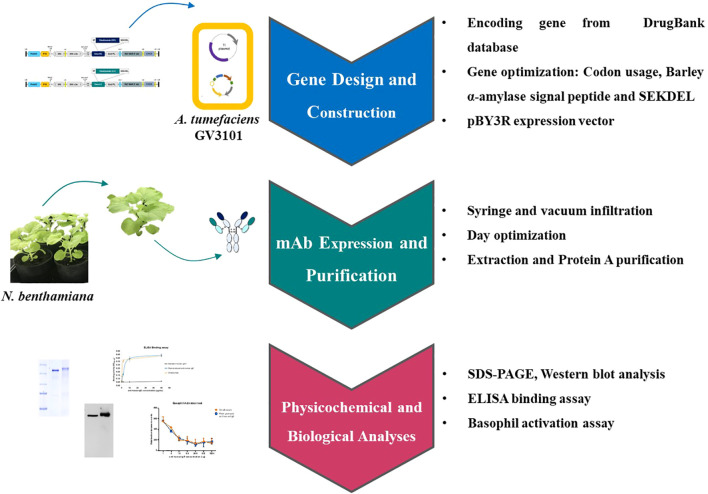

## Introduction

Asthma is characterized as a chronic inflammatory condition of the respiratory tracts that can relapse frequently with severity overtime. Most respiratory symptoms include attacks of breathlessness, coughing, wheezing with fluctuating expiratory airflow limitation. Asthma is a well-known chronic, noncommunicable disease (NCD), affecting both children and adults with a approximate total of 300 million individuals globally (Ali et al., 2020; WHO, 2021). In brief, allergic reactions begin with allergen exposure to antigen-presenting cells (APCs) such as macrophages and dendritic cells mostly, where they are processed and presented to the specific peptide epitope on their cell surface. A naïve CD4^+^ T-cell lymphocyte responds to the presented epitope driving the T cells to become T-helper (T_H_2) cells, which further release interleukins IL-4/IL-5/IL-13 and interact with receptors on cell surface of the B lymphocyte. This interaction plays the central role in inducing the B cell class switching to immunoglobulin E (IgE) (Takhar et al., 2007). IgE is the hallmark in mediating an allergic hypersensitivity reaction *via* interaction of the IgE-Fc region with its two receptors (Pennington et al., 2016). The first receptor is the high-affinity IgE receptor (FcϵRI) expressed on the surface of basophils and mast cells as tetramers and APCs as trimers at very low levels. The second is the low-affinity IgE receptor (FcϵRII or CD23) present on the surface of B cells and other hematopoietic cells, which is capable of IgE regulation and antigen presentation (Stone et al., 2010; Liu et al., 2020) (Belliveau, 2005). Crosslinking of the allergen with IgE/FcϵRI complexes trigger mast cell degranulation and release the inflammatory mediators such as histamine, heparin, tryptase, chymase, arachidonic acid metabolites, prostaglandins, leukotrienes, etc. resulting in allergic symptoms such as mucous hypersecretion, bronchial thickening and edema (Belliveau, 2005; Thomson and Chaudhuri, 2012; Nguyen et al., 2021).

IgE-targeted therapy becomes a key driver in the treatment of asthma, attempting to lower the level of free IgE in blood and prevent degranulation of granulocytes (mast cells, basophils), following the binding of IgE to high-affinity IgE receptors on mast cells and basophils. Biologic-targeted treatment has been successfully introduced as an add-on therapy or controller medications to improve asthma condition in patients with moderate-to-severe persistent allergic asthma and chronic spontaneous urticaria (Global Initiative for Asthma, 2022). Few monoclonal antibodies are recommended for asthma treatment include Omalizumab (anti-human IgE), Mepolizumab (anti-IL-5/IL-5R), and Dupilumab (anti-IL-4R).

Omalizumab (Xolair), is an anti-human IgE monoclonal antibody (mAb) commercialized in the market worldwide as an add-on therapy for patients with severe allergic asthma and medicaments with inhaled corticosteroids (ICS) and long-acting β2-agonists (LABA) (Global Initiative for Asthma, 2022). It is a recombinant humanized anti-IgE monoclonal antibody with human immunoglobulin G (IgG) configuration containing 5% murine amino acid sequences at complementarity-determining regions (CDRs) and 95% human sequences. Omalizumab acts by hindering the binding of IgE to FcϵRI receptor thereby, not allowing the progression of allergic inflammations (Lin et al., 2004). It neutralizes the free IgE by binding at the Cϵ3 domain of Fc fragment where the high affinity receptor (FcϵRI) binds thus reducing their levels in serum (Lowe and Renard, 2011; Nguyen et al., 2021). The reduction of free IgE circulation in the blood stream leads to down-regulation of FcϵRI receptor on effector cells (Macglashan et al., 1997; Saini et al., 1999; Macglashan and Saini, 2013).

Omalizumab was recombinantly expressed in the suspension cell culture of Chinese Hamster Ovary (CHO) cell line. Mammalian cells have traditionally been used as the preferred host for mAb expression because they can produce antibodies that are almost identical to those found in the human body while also posing the least risk of non-human glycosylation patterns (Shanmugaraj et al., 2020a). However, this system has limitations such as high risk of human pathogen contamination and high production costs requiring controlled aseptic manufacturing environment (Frenzel et al., 2013). In this study, we investigated the feasibility of production of anti-human IgE mAb employing plants as expression platform. The plant-based expression system has been utilized for the production of P2G12 mAb (Ma et al., 2015), Trastuzumab (Mclean, 2017), Denosumab (Boonyayothin et al., 2021). This platform also offers other advantages, including lower production cost, the lack of the human pathogen contamination and its ability to provide post-translational modification (PTMs) which is crucial for structure and functional integrity (Fischer et al., 2004; Shanmugaraj et al., 2020a). The present study aims to develop *Nicotiana benthamiana* plants as an expression platform for producing the anti-human IgE mAb. The main objective was to express and characterize the plant-based anti-human IgE that was compared with commercial Omalizumab (Xolair) using SDS-PAGE, binding by ELISA and basophil activation assay.

## Materials and methods

### Reagents, enzymes, antibodies

The pBY3R geminiviral expression vector was provided by Professor Hugh Mason (Arizona State University, Tempe, Arizona, USA). Taq DNA polymerase used for polymerase chain reaction (PCR) was a product of Vivantis Technologies (Selangor, Malaysia). Restriction endonucleases (*Bsa*I, *Nhe*I, *Afl*II, *Xba*I, and *Sac*I) and T4 DNA ligase were purchased from New England Biolabs (Hitchin, UK). Standard native human IgG1 was purchased from Abcam (Cambridge, United Kingdom). Amintra^®^ Protein A resin for antibody purification was a product of Expedeon (Cambridge, UK). All blue pre-stained protein standard and nitrocellulose membrane (0.45 micron) were bought from BioRad (US). Coomassie brilliant blue was purchased from Applichem (Darmstadt, Germany). Anti-human kappa antibody was purchased from Southern Biotech (Alabama, US) and anti-human gamma antibody was purchased from The Binding Site (Birmingham, UK). Amersham ECL prime western blotting detection reagent was a product of GE Healthcare (Illinois, US). Humanized Rat Basophilic Leukaemia cell line (RS-ATL8) used for IgE cross-linking-induced luciferase expression was kindly provided by Prof. Tanapat Palaga, Department of Microbiology, Faculty of Science, Chulalongkorn University. Other chemical reagents were of analytical grade.

### *Nicotiana benthamiana* plant growth conditions

Wild-type *N. benthamiana* plants were cultivated in a controlled environment in the greenhouse. The plants were grown in pots containing a soil mixture of peat moss, vermiculite, and perlite in the ratio of 4:2:1 (Ji et al., 2018). The greenhouse was maintained at a temperature of 25 ± 2°C with 16 ± 8 hours of day night cycle, light intensity 80–100 μmol/m^2^s^− 1^ and around 60% humidity. The plants were grown for 4-6 weeks before agroinfiltration.

### Construction of expression vector with anti-IgE heavy chain and light chain

The gene fragments encoding the variable coding regions of the heavy chain (HC) and light chain (LC) of the Omalizumab (Drugbank accession number: DB00043) and human IgG_1_ constant region (Huang et al., 2010) sequences were retrieved for the construction of expression vector. The variable region of heavy chain and light chain sequences were codon optimized *in silico* for expression in *N. benthamiana* plants using GeneArt GeneOptimizer software (Invitrogen, Thermo Fisher Scientific, MA, United States). The codon-optimized sequences were synthesized at Biomatik, Canada. The variable regions in the heavy and light chain genes of anti-IgE antibody synthesized were digested using *Bsa*I/*Nhe*I and *Bsa*I/*Afl*II restriction enzymes, respectively and the digested DNA products on agarose gel electrophoresis were further purified by using Accuprep PCR/gel purification kit (Bioneer, Korea). For the heavy chain, the variable (V_H_) domain was ligated with constant heavy chain domain (C_H_1-C_H_2-C_H_3), in the pBY3R geminiviral expression vector using T4 DNA ligase (New England Biolabs, Hitchin, UK). For the light chain, the variable (V_L_) domain was ligated with constant (C_L_) regions in pBY3R expression vector. The HC and LC encoding DNA sequences were cloned with the endoplasmic reticulum (ER) retention signal peptide SEKDEL (Ser-Glu-Lys-Asp-Glu-Leu) at the carboxyl terminus (C-terminus) to generate full length HC and LC gene sequences.

In this study, the geminiviral replicon system derived from the bean yellow dwarf virus (Chen et al., 2011) was used for rapid production of anti-human IgE mAb. The positive recombinant plasmid was confirmed by restriction enzyme analysis using *Xba*I and *Sac*I. Then the *A. tumefaciens* strain GV3101 was separately transformed with the Omalizumab-heavy chain expression vector pBY3R (pBY3R-Oma-HC) and Omalizumab-light chain (pBY3R-Oma-LC) genes by electroporation. The transformed *A. tumefaciens* cells were cultured in lysogeny broth media (supplemented with rifampicin, kanamycin and gentamicin at a final concentration of 50 µg/L) and confirmed using PCR with gene-specific forward and reverse primers (PCR cycling condition: an initial denaturation at 96°C for 5 min, followed by 35 cycles of 96°C for 30 s, 52°C for 30 s, and 72°C for 1.5 min, and a final extension at 72°C for 10 min). The positive clones of transformed *A. tumefaciens* were chosen for further experiments.

### Generation of plant-produced anti-IgE antibody

Plant-derived anti-human IgE mAb was transiently expressed in *N. benthamiana* using the geminiviral vector. Recombinant *A. tumefaciens* harboring the plant expression vector containing either HC or LC were inoculated in lysogeny broth supplemented with rifampicin, gentamicin, and kanamycin at a final concentration of 50 mg/L. The cultures were grown in an incubator shaker at 28°C with 200 rpm overnight. The overnight bacterial culture medium was collected and harvested by centrifuging at 1000 g for 10 min. The cell pellet was gently washed and resuspended in infiltration buffer [10 mM 2-*N*-morpholino-ethanesulfonic acid (MES), pH 5.5 and 10 mM MgSO_4_] to obtain a final optical density (OD_600_) of 0.4 at 600 nm. Agrobacterial suspension containing HC and LC were mixed together in the ratio of 1:1 and co-infiltrated into 30-35 day-old wild-type *N. benthamiana* leaves by syringe infiltration whereas the large-scale infiltration was performed by vacuum infiltration. After infiltration, the plants were returned to the greenhouse with the controlled condition (temperature at 25 ± 2°C with 16-h light/8-h dark cycle) before being harvested.

### Screening of days post-infiltration for high anti-IgE mAb expression

For time-course experiment of plant-produced anti-human IgE mAb expression levels in plants, infiltration buffer containing the recombinant pBY3R-Oma-HC and pBY3R-Oma-LC (ratio 1:1) were infiltrated into plant leaves. The infiltrated leaves were collected on different days post-infiltration (dpi) that include 2, 4, 6, 8, and 10 dpi to estimate the optimal incubation time that can express high yields of plant-produced anti-human IgE. The infiltrated leaves were ground in liquid nitrogen using a mortar and pestle. The powdered leaf was added with 1:2 w/v cold extraction buffer (Phosphate buffered saline (1X PBS); 137 mM NaCl, 2.68 mM KCl, 10.1 mM Na_2_HPO_4_, and 1.76 mM KH_2_PO_4_, pH 7.4) and vortexed for 5 mins The clarified crude extracts were spun down by centrifugation at 26000 g for 30 min at 4°C. The supernatants were used to measure the total soluble protein (TSP) using Bradford assay (Bio-Rad, USA) following manufacturer’s instructions. The clarified crude extract was further analyzed by SDS-PAGE and Western blot analysis.

### Purification of plant-derived anti-human IgE mAb

The harvested leaves after agroinfiltration were collected and extracted in extraction buffer (1X PBS, pH 7.4) using an electric drill. Supernatants were collected by centrifugation at 26000 g for 30 min and 0.45µm filtration (Millipore Sigma, United States) was performed to clarify plant crude extract containing anti-IgE mAb. For purification, the anti-human IgE mAb in the clarified crude extract was specifically captured by Protein A affinity chromatography. Briefly, 1.0-1.5 mL of Amintra Protein A resin was packed into a 10 mL polypropylene column to a bed height of 1 cm and equilibrated with 5 column volumes (CV) of the extraction buffer (1X PBS, pH 7.4) at a flow rate of 2 mL/min. After column equilibration, the clarified crude extracts were applied at a flow rate of 1 mL/min and the wash step was performed with 10 CV of wash buffer (1X PBS, pH 7.4). Elution was done at a flow rate of 1 mL/min using 5 mL of elution buffer (0.1 M glycine, pH 2.7) with an incubation time of 5 mins prior to the collection of each 1 mL. The elutes were neutralized with 1.5 M Tris-HCl (pH 8.8). The neutralized elutes were desalted and concentrated with Amicon ultra-centrifugal filter (Merck, Massachusetts, United States) in 1X PBS buffer (pH 7.4). The protein’s structure integrity in the purified mAb was confirmed by Sodium dodecyl sulfate-polyacrylamide gel electrophoresis (SDS-PAGE) and Western blotting analysis under reducing and non-reducing conditions.

### Identification of anti-IgE mAb by SDS-PAGE and western blot

The protein structure integrity and product-related variants were characterized using SDS-PAGE and Western blot analysis. Commercial anti-human IgE mAb (Omalizumab; Xolair, Novartis, United States) was used as positive control. The samples were analyzed under reducing and non-reducing conditions. For non-reducing conditions, the protein samples were mixed with SDS-loading buffer [125 mM Tris-HCl, pH 6.8, 12% w/v SDS, 10% v/v glycerol, and 0.001% (w/v) bromophenol blue]. The protein sample denatured at 95°C for 5 min was separated on 12% freshly prepared polyacrylamide gels. For reducing conditions, the sample was added to SDS-loading buffer containing 22% β-mercaptoethanol, denatured at 95°C for 5 min, and then separated on 12% polyacrylamide gels. The gels were stained with Coomassie brilliant blue (AppliChem, Germany), and the bands were visualized.

For Western blot analysis, the separated protein samples were transferred on to nitrocellulose membranes (Bio-Rad, USA). The nitrocellulose membranes were blocked with 5% skim milk in 1X PBS (pH 7.4) for 1 h at room temperature of 25 ± 2°C. The membranes were washed thrice with PBS before incubation with either a 1:5,000 dilution (0.2 mg/L) of HRP-conjugated anti-human gamma antibody (The binding site, UK) or a 1:5,000 dilution (0.1 mg/L) of HRP-conjugated anti-human kappa antibody (Southern Biotech, USA) in 3% skim milk prepared in 1X PBS (pH 7.4). After incubation, the membrane was washed thrice with 1X PBST, and the bound antibody was detected using Amersham ECL prime western blotting detection reagent (GE Healthcare, UK) and the signal was captured on medical X-ray green (MXG) film (Carestream, USA).

### Binding profile of anti-human IgE mAb by ELISA

The binding efficiency of plant-produced anti-human IgE mAb with IgE was determined using enzyme-linked immunosorbent assay (ELISA). 5 µg/mL human IgE was coated onto a 96-well ELISA plate (Griner Bio One GmbH, Austria) and incubated at 37°C for 4 h. Then, the plate was blocked with 200 µL of 5% skim milk (BD, Franklink Lakes, NJ, United States) in 1X PBS (pH 7.4) at 37°C for 2 h and washed three times with 1X PBST. Various concentrations of plant-produced anti-human IgE, commercial Omalizumab (positive control) were diluted in PBS and added to triplicate wells followed by incubation at 37°C for 2 h. After washing three times with 1X PBST (1X PBS containing 0.05% Tween-20), the plate was incubated at 37°C for 1 h with HRP-conjugated anti-human gamma IgG1 antibody (The Binding site, United Kingdom) at a dilution of 1:1,000 in 1X PBS. The plate was developed by adding SureBlue TMB 1-Component Microwell Peroxidase Substrate (Promega, United States), and the reaction was stopped by 1 M H_2_SO_4_. The absorbance was measured using a microplate reader at an optical density of 450 nm (OD_450_).

### IgE cross-linking induced luciferase expression study

The RS-ATL8 cells, humanized rat basophilic leukemic cells, were cultured in minimum essential medium (MEM) supplemented with 10% fetal bovine serum (FBS), 1% Penicillin/Streptomycin, 0.5 µg/mL geneticin, 0.2 µg/mL hygromycin B, and GlutaMAX-I. All the reagents for RS-ATL8 cell culture were procured from Gibco; ThermoFisher Scientific, USA. Measurement of luciferase activity was performed following the procedures described previously (Nakamura et al., 2012; Gericke et al., 2015; Jarupalee et al., 2018) with some modifications. Briefly, the RS-ATL8 cells were seeded at 5×10^4^ cells per well in 96 well-plate (50 μL per well) and incubated for 3 h. Cells were then sensitized overnight with the pre-mixture of 5 μg/mL of human-IgE (Abcam, USA) and various concentrations (0.5-512 μg/mL) of plant-produced anti-human IgE (stock 3.4 mg/mL) or Omalizumab (stock 75 mg/mL) diluted in PBS, for 1 h at 37°C. After sensitization, cells were washed once gently with sterile PBS to remove the unbound antibodies. The pre-mixtures that contain only human IgE and only 512 μg/mL of plant-produced anti-human IgE or Omalizumab were used as positive and negative control, respectively. The sensitized cells were then crosslinked with 5 μg/mL of goat anti-human IgE (Millipore, USA). After 3 h of cross-linking, luciferase activity was measured by the ONE-Glo Luciferase Assay System (Promega Corporation, Madison, WI, USA) using a Varioskan microplate reader (ThermoFisher Scientific, Vantaa, Finland) with luminometric mode. The relative luciferase unit (RLU) was calculated by subtracting the signal from the negative control wells. The study was performed in duplicate wells for three independent runs and the average was used for analysis and the values were expressed as mean ± SD.

## Results

### Transient expression and purification of plant-derived anti-human IgE in *N. benthamiana*

**Figure 1:**
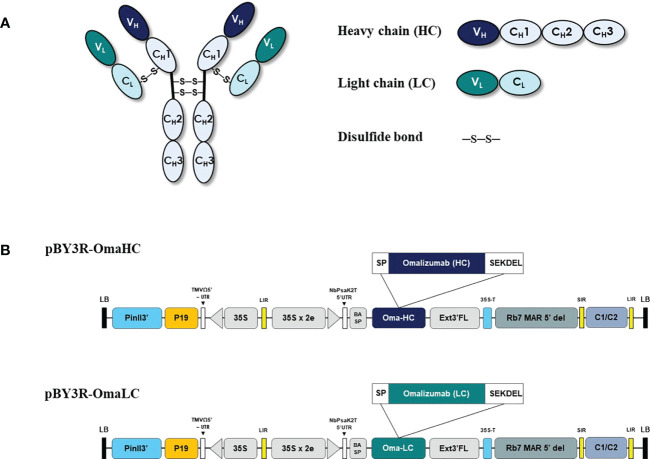
Schematic representation of pBY3R vector used in the present study for expression of anti-human IgE antibody (Omalizumab). **(A)** Schematic and structural elements of heavy chain (HC), light chain (LC) of codon optimized Anti-IgE antibody for expression in plant host and assembled plant produced anti-IgE mAb. **(B)** T-DNA region of the pBY3R vector; P35S: cauliflower mosaic virus (CaMV) 35S promoter; NbPsaK2T 5**’**UTR: 5**’** untranslated region; BASP: barley α-amylase signal peptide; Oma-HC: anti-IgE antibody heavy chain; Oma-LC, anti-IgE antibody light chain; Ext3**’** FL: Full length of the tobacco (*Nicotiana tabacum*) extension gene; 35 S-T: CaMV 35S terminator l; Rb7 MAR 5**’**del: tobacco RB7 promoter; SIR: short intergenic region of BEYDV genome; LIT: long intergenic region of BeYDV genome; C2/C1: bean yellow dwarf virus (BeYDV) open reading frames C1 and C2 encoding for replication initiation protein (Rep) and RepA; P19: P19 gene from tomato bushy stunt virus (TBSV); PinII 3**’**: terminator from potato proteinase inhibitor II gene.

**Figure 2:**
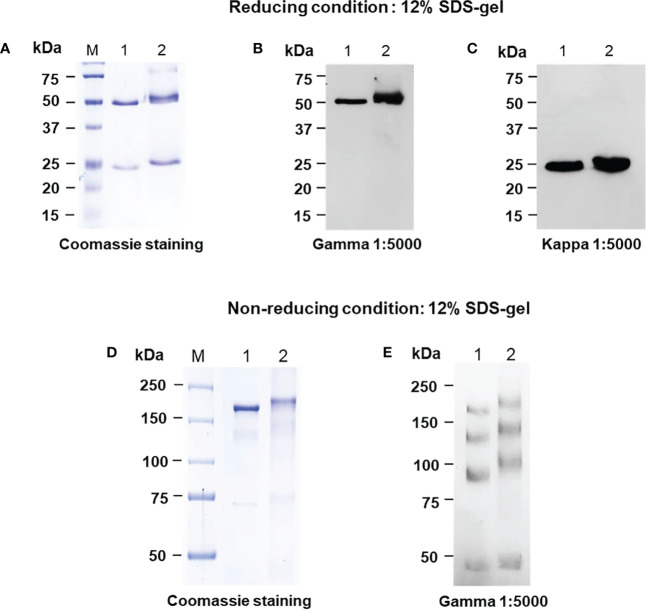
SDS-PAGE analysis of the purified plant-produced anti-human IgE antibody in *Nicotiana benthamiana* under reducing and non-reducing conditions using SDS-PAGE **(A, D)** and Western blot analysis with either HRP-conjugated goat anti-human gamma chain antibody **(B, E)** or goat anti-human kappa chain antibody **(C)**. 150-450 ng of the plant purified protein and commercial Omalizumab was loaded in the well. Lane M: Protein ladder, Lane 1: Commercial Omalizumab (Xolair), Lane 2: Purified plant-produced anti-human IgE antibody.

For the anti-IgE antibody production in plant host *N. benthamiana*, the variable regions of heavy and light chain genes of Omalizumab were fused with human IgG_1_ along with signal peptide and SEKDEL sequences at N-C terminals respectively and were cloned into pBY3R geminiviral expression vector (**Figure 1**) and subsequently electroporated into *A. tumefaciens* strain GV3101. *Agrobacterium* harboring the Omalizumab genes were co-infiltrated into the *N. benthamiana* plant leaves to further evaluate the expression of anti-IgE antibody. The infiltrated leaves were harvested at specific timepoints, extracted, and purified for the anti-human IgE antibody by Protein-A affinity chromatography. Further, the purified anti-human IgE antibody was analyzed using SDS-PAGE (under both reducing and non-reducing conditions) stained with Coomassie brilliant blue stain to visualize the antibody fragments and assembling of the purified plant-produced anti-IgE mAb. Western blot analysis was performed to confirm the purified plant-produced mAb by probing with anti-human gamma-HRP and anti-human kappa-HRP. As shown in **Figure 2**, the SDS-PAGE profile under reducing condition (**Figure 2A**) showed protein bands at molecular sizes of approximately 50 and 25 kDa respectively corresponding to the heavy and light chains of the antibody, whereas the non-reducing condition showed bands at 150 kDa (**Figure 2D**) in similar to the commercial Omalizumab.

The protein bands were further confirmed by western blot using specific goat anti-human kappa and goat anti-human gamma IgG antibodies antibody. As presented in **Figures 2B, C, E**, the western blot results showed a detectable signal, confirming the expression of target protein. The results from western blot analysis were correlated with SDS-PAGE, confirming that the protein bands (both HC and LC) observed are at an approximate molecular weight of 50 and 25 kDa respectively (**Figures 2B, C**). Light chains interact with heavy chains and heavy chains can also interact with each other. The recombinant mAbs can exist as combination of heavy and light chains and mostly the F(ab’)_2_ fragments exhibit multiple unfolded states in non-reducing SDS (Kiessig et al., 1992). This could be the possible reason for multiple bands in both plant-produced and commercial Xolair (**Figure 2E**).

### Analysis of anti-IgE expression in *N. benthamiana*

**Figure 3:**
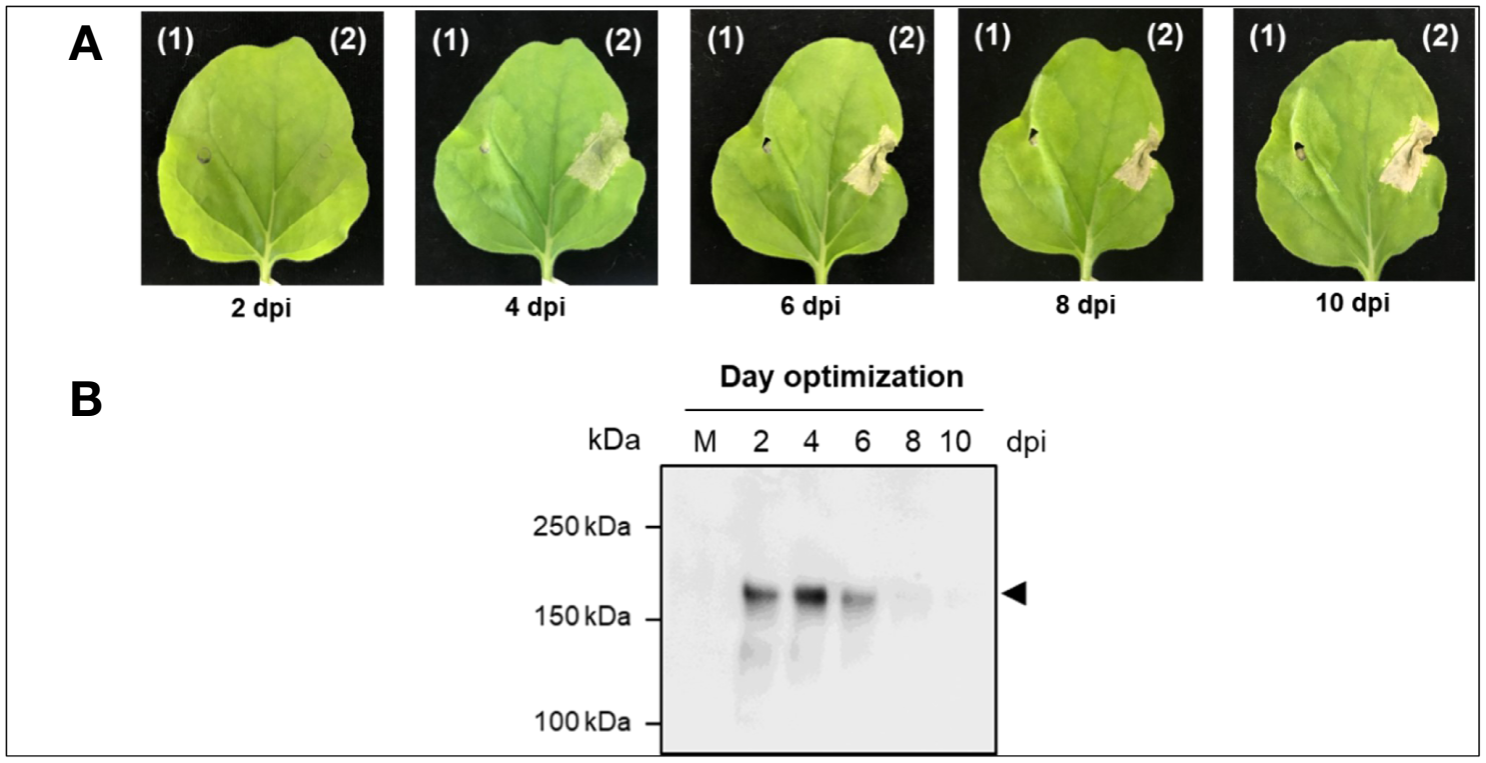
Transient expression of Anti-IgE antibody in *N. benthamiana* plants **(A)** Typical phenotype of *N. benthamiana* leaves expressing anti-human IgE mAb on 2, 4, 6, 8, and 10 dpi. **(B)** Representative non-reducing western blot analysis of crude extract expressing anti-human IgE mAb on different dpi (2, 4, 6, 8, and 10). The protein was transferred on to a nitrocellulose membrane and the blot was probed with HRP-conjugated anti-gamma antibody. kDa: kilodalton; dpi: days post-infiltration; the lane number represents the harvested day post-infiltration expressing plant-produced anti-human IgE mAb. Arrowhead indicates the expression of full-length anti-human IgE mAb in plant leaves.

Preliminary studies with anti-IgE gene constructs having with and without SEKDEL at C-terminus were performed to determine the expression levels of anti-IgE mAb in *N. benthamiana.* The anti-IgE construct with SEKDEL demonstrated higher anti-IgE accumulation (**Supplementary figure S1**). The anti-IgE antibody expression with the SEKDEL gene construct was screened on different days after post-infiltration on 2, 4, 6, 8, and 10. The infiltrated leaves were harvested on different days and the expression level was detected by western blot analysis. As shown in **Figure 3**, the highest expression of plant-produced anti-human IgE from infiltrated leaves was apparently observed with intense band on 4 dpi with the expression yields up to 41.2 µg/g of leaf fresh mass after subsequent steps of purification. The onset of necrosis was observed in the infiltrated plant leaves on day 4 post infiltration and increased in the following days of 6, 8 and 10. Hence, the 4 dpi was chosen for further analysis. The significant difference in the expression levels on different days post infiltration was not established by statistical methods, owing to the small sample size used in the study.

### Binding assay of anti-IgE antibody by ELISA

**Figure 4:**
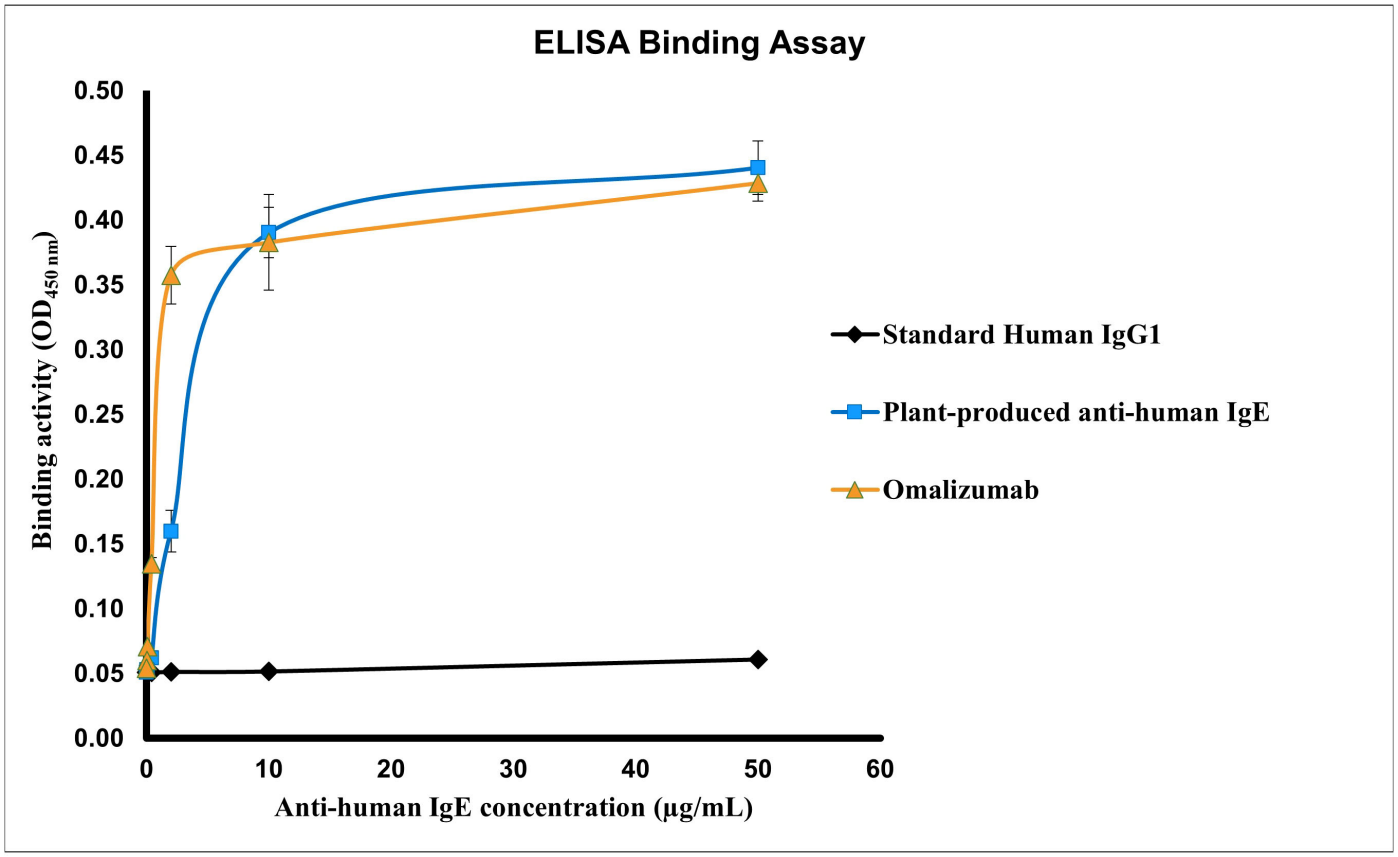
Specific binding activity of the purified plant-produced anti-human IgE mAb to human IgE. Various concentrations ranging from 0.016-50 µg/mL of the anti-human IgE mAb were analyzed for the assessment of binding activity. The protein samples used in the assay include the purified plant-produced anti-human IgE, standard human immunoglobulin 1 (IgG1) (negative control) and commercial Omalizumab (positive control). Data are expressed as the mean ± SD of triplicates.

The binding efficiency of plant produced anti-IgE was determined by ELISA as depicted in **Figure 4**. The interaction of the anti-human IgE mAb with its target binding site on IgE was assessed in this assay, which is the prime mode-of-action of Omalizumab in allergic conditions (Guntern and Eggel, 2020). Both plant produced and commercial Omalizumab showed similar binding to human IgE target receptors in all concentrations (0-50 µg of each antibody) analyzed. This implicates the functional ability of the plant expressed anti-IgE mAb. The dissociation constant (K_d_) was found to be 14.8 µg/mL and 0.9043 µg/mL for plant produced anti-IgE and commercial Omalizumab respectively, using GraphPad Prism 8.1.2 (GraphPad Software, Inc. La Jolla, CA). Human IgG_1_ that was used as the negative control could not bind to the human IgE receptor protein.

### Inhibition of IgE cross-linking in RS-ATL8 reporter cells

**Figure 5:**
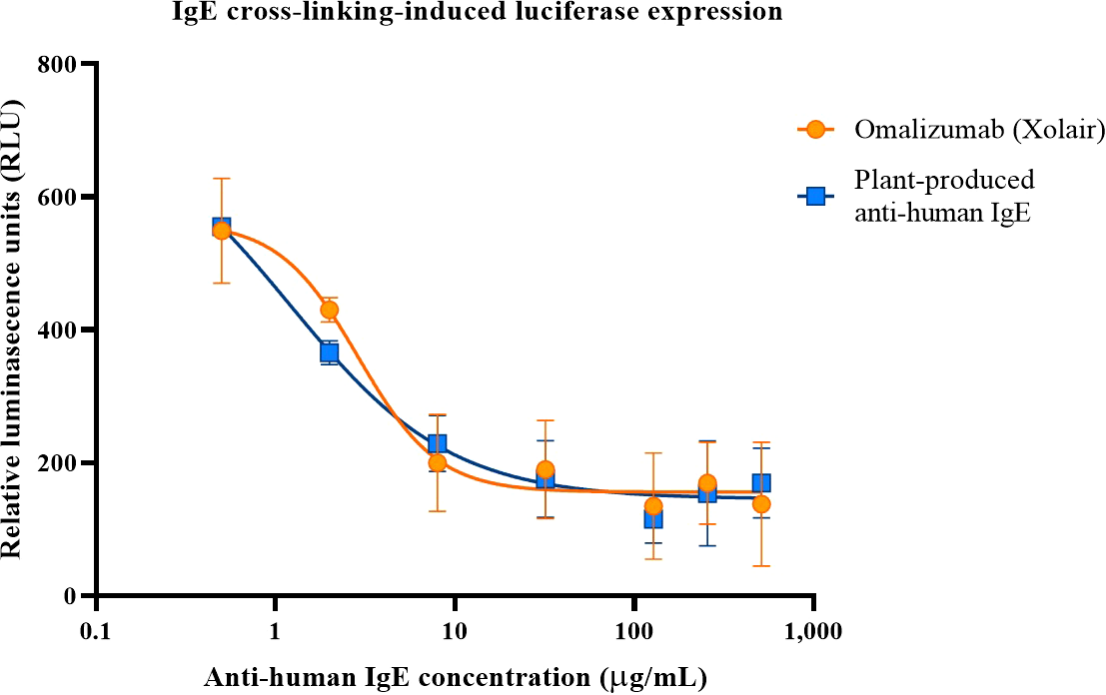
Inhibition of IgE-crosslinking-induced luciferase expression in RS-ATL8 reporter cells. RS-ATL8 cells were sensitized overnight with pre-mixture of human IgE and various concentrations of Omalizumab (Xolair)/orange or plant-produced anti-human-IgE/blue. After sensitization, goat anti-human IgE was added to allow crosslinking. Luciferase expression level after 3 h of crosslinking was analyzed. Data are expressed as mean ± SD of the duplicated-well readings of 3 independent experiments. The difference in RLU generated by plant produced anti-human-IgE and Xolair was analyzed by Mann-Whitney test (p > 0.05) in every concentration.

Determination of plant produced anti-human IgE to inhibit IgE cross-linking-induced luciferase expression using RS-ATL8 cell line was performed. The results revealed that plant-produced anti-human-IgE could inhibit IgE crosslink in a dose-dependent manner (**Figure 5**). The reduction of relative luminescence units (RLU) generated by plant produced anti-human-IgE was found to be comparable with commercial Omalizumab in every concentration analyzed. The minute difference in RLU generated by plant produced anti-human-IgE and Xolair was analyzed by Mann-Whitney test (p > 0.05) in every concentration. The 50% inhibitory concentration (IC50) for plant produced anti-human-IgE and commercial Omalizumab were 1.208 and 2.887 µg/mL, respectively. This could be confirmed that the plant-produced anti-human IgE exhibited the bioactivity and function, at least, similar to those of commercial Omalizumab (Xolair).

## Discussion

Anti-IgE therapy acts as an essence in the care and treatment of allergic respiratory symptoms and still needs further investigation for unravelling its salient features for application in several other IgE mediated complex disorders (Pennington et al., 2016; Pelaia et al., 2018; Henriksen et al., 2020). Omalizumab was the only biologic drug approved as additional treatment for chronic asthmatic conditions (Pelaia et al., 2018). Although, the Omalizumab currently available in markets illustrate good safety, efficacy and tolerability (Tonacci et al., 2017; Pelaia et al., 2018; Henriksen et al., 2020) in asthma patients, the expenses for one year treatment ranges approximately from $20,000 to $32,000 as mentioned by Sims, 2004 (Sims, 2004) depending on the disease severity and body weight of the individual. Hence, an alternative has to be developed to express the recombinant Omalizumab for therapeutic use in a cost-effective way.

Over the last few decades, abundant literature reveals that plant-based expression of recombinant proteins is a promising technology (Shanmugaraj and Phoolcharoen, 2021) and was successful in the expression of virus-like-particles (VLP’s) (Ward et al., 2021), monoclonal antibodies (Shanmugaraj et al., 2020b; Swope et al., 2022), antigens (Rattanapisit et al., 2020b), growth factors (Bulaon et al., 2020; Hanittinan et al., 2020; Rattanapisit et al., 2020a), enzymes (Fox, 2012) due to the advantages in terms of high reproducibility, scalability in short time with lower contamination menace (Shanmugaraj et al., 2020a). Accordingly, the present study focuses on the recombinant production of anti-IgE antibody by *Agrobacterium* mediated transformation in *N. benthamiana* host system. The retrieved heavy and light chain sequences from drug bank were codon optimized, synthesized, and constructed into the geminiviral plant expression vector (**Figure 1**). The heavy and light chains were ligated with the signal peptides, barley alpha-amylase (Hunter et al., 2019) and endoplasmic retention SEKDEL (Petruccelli et al., 2006) sequence at N- and C- terminals respectively to enhance the localized expression of the anti-IgE mAb. Endoplasmic reticulum organelle based anti-IgE antibody expression was targeted because of the presence of exclusively non-immunogenic high mannose type N-glycans as reported elsewhere (Sriraman et al., 2004; Petruccelli et al., 2006). Additionally engineering the recombinant proteins with KDEL can enhance accumulation levels, alter solubility, or facilitate recovery with no apparent adverse effect of the ER-retention signal on stability and activity of the target proteins (Liénard et al., 2007; Galpin et al., 2010). Also the C-terminal ER-retention signal KDEL mediate the retrieval of proteins from a post-ER compartment back to the ER lumen by a retrograde transport (Murshid and Presley, 2004) and may not be subject to N-glycan maturation when synthesized in plant cells (Galpin et al., 2010).

The present study results demonstrate that the plant-produced anti-human IgE mAb can be successfully assembled into a native IgG monomeric form (**Figure 2D, E**) with the highest expression of 41.2 µg/g fresh mass obtained at 4 dpi in *N. benthamiana* (**Figure 3B**), when compared to the anti-IgE mAb infiltrated leaf samples harvested on 2, 6, 8 and 10 dpi. Previous studies reported the maximum yields of various transiently expressed proteins such as pembrolizumab (Phakham et al., 2021), B38, H4 mAb’s (Shanmugaraj et al., 2020b), SARS-CoV-2 RBD (Siriwattananon et al., 2021) after 4 dpi, 2G12 mAb (Buyel and Fischer, 2012), viscumin A (Gengenbach et al., 2019) after 5 dpi and anti-PD1 mAb (Rattanapisit et al., 2019) on 6 dpi in *N. benthamiana.* The study implies the benefit of transient expression over stable transgenic plant expression (Ma et al., 2015) and mammalian expression (Dalton and Barton, 2014) in terms of reduced protein production timeline, scalability using low cost upstream process and adaptability (Shanmugaraj et al., 2020a). Furthermore, the optimal incubation day might be different based on *Agrobacterium* strain used, protein characteristics, agroinfiltration procedures that have been tested with modifications such as addition of chemical additives in the infiltration buffer, application of heat shock after agroinfiltration, and the co-expression of suppression gene to enhance the transient gene expression (Norkunas et al., 2018). In addition, as presented in **Figure 3A**, the occurrence of leaf necrosis apparently starts on 4 dpi, due to tissue damage caused by infiltration or the defense/hypersensitive response triggered by the recognition of bacteria by pathogen receptors (Buyel et al., 2015) and endoplasmic reticulum stress due to protein accumulation. This event is considered as a crucial factor for decreased protein yields (Mathew et al., 2014; Diamos and Mason, 2019). However, many reports have shown the promising advantages in targeting the protein expression to the ER compartment in terms of increased protein accumulation and protein quality (Petruccelli et al., 2006; Benchabane et al., 2008). Affinity chromatography was used to purify the recombinant anti-IgE mAb from plant crude extracts. As evident from the visual inspection of coomassie-stained SDS gel (**Figure 2B**), the purity of the anti-IgE mAb was achieved up to 80% with more than 60% recovery by using single-step Protein-A purification. However, purification process needs to be further refined to get rid of process related impurities, cell debris, host cell proteins, high molecular weight aggregates and nicotine in the purified anti-IgE mAb (Swope et al., 2022).

The binding activity of plant produced anti-IgE mAb was further assessed by immunoassay, which showed similar interaction with IgE in comparison with commercial Omalizumab (Xolair). The difference in the binding properties may be attributed due to purity of the target protein and interference of process related impurities. Although N-glycosylation was not evaluated, glycosylation of antibodies is carried out in a different manner in plant cells than in mammalian cells (Cabanes-Macheteau et al., 1999; Bakker et al., 2001) modifying the biological activities of antibodies (Ko, 2014). Antibodies like nivolumab (Rattanapisit et al., 2019), pembrolizumab (Phakham et al., 2021), rituximab (Bennett et al., 2018) expressed in plants have similar binding capacities as the ones expressed in mammalian cells (Liénard et al., 2007; Ko, 2014). Moreover, the bioactivity of plant produced anti-IgE mAb was further studied by the IgE cross-linking induced plant produced anti-IgE in RS-ATL cell lines, also known as basophil activation assay. As shown in **Figure 5**, the plant produced anti-IgE was efficient in blockage of the activation of basophil, mediated by IgE crosslinking which is comparable to Omalizumab (Xolair). This was initially proved that anti-IgE produced in our plant expression system was feasible in production of functional mAb similar to the commercial one. However, further investigations including the binding affinity as well as *in vivo* study in animal models are warranted for further exploration. In summary, this study successfully expressed and purified anti-human IgE mAb in *N. benthamiana* indicating that *Nicotiana*-based system is efficient to produce and support the development of other biopharmaceutical products.

## Conclusion

A gradual increase in the prevalence of IgE-mediated diseases including the asthma has put an insight to scrutinize, develop and produce anti-IgE antibody. Although, the anti-IgE mAb namely Omalizumab is commercially available in the market, it involves high costs that cannot be affordable by the middle- and low-income countries. The present study aimed at producing anti-IgE antibody using plant as an expression host. The plant produced anti-IgE antibody expression was optimized and further purified by protein A chromatography, analyzed by SDS-PAGE and western blot. The binding and functionality of the plant produced anti-IgE antibody was compared with commercially available Xolair. However, we acknowledge that there is a need to refine the purification approach for enhancing the protein yield and improve the product quality attributes to meet the industry standards. Further studies shall be performed in animal models to test the safety, efficacy and evaluate whether the plant produced anti-IgE antibody may benefit in clinical application and inexpensive therapeutic treatment of IgE mediated diseased patients.

## Data availability statement

The raw data supporting the conclusions of this article will be made available by the authors, without undue reservation.

## Author contributions

WP conceived the study. OH and KR performed the cloning, purification, quantification studies, analyzed the results and drafted the manuscript. AM participated in literature search, edited, and reviewed the manuscript. KT performed the *in vitro* functional study. CK conceptualized the *in vitro* study. EP designed, conceptualized the *in vitro* study, and drafted the functional study part in the manuscript. All authors revised the manuscript and approved the final manuscript for submission.

## Funding

This Research is funded by Thailand Science Research and Innovation Fund Chulalongkorn University (CU_FRB65_hea (55) 064_33_08). The funder was not involved in the study design, collection, analysis, interpretation of data, the writing of this article or the decision to submit it for publication.

## Conflict of interest

WP is a co-founder/shareholder of Baiya Phytopharm Co., Ltd. Authors KR and AM have potential financial competing interest due to paid employment provided by Baiya Phytopharm Co., Ltd.

The remaining authors declare that the research was conducted in the absence of any commercial or financial relationships that could be construed as a potential conflict of interest.

## Publisher’s note

All claims expressed in this article are solely those of the authors and do not necessarily represent those of their affiliated organizations, or those of the publisher, the editors and the reviewers. Any product that may be evaluated in this article, or claim that may be made by its manufacturer, is not guaranteed or endorsed by the publisher.

## Supplementary material

Supplementary Figure 1 Effect of SEKDEL signal peptide on the anti-IgE antibody expression in *N. benthamiana* plants. **(A)** Typical phenotype of *N. benthamiana* leaves expressing anti-human IgE mAb with or without SEKDEL on 2, 4, 6, 8, and 10 dpi; **(B)** Representative non-reducing western blot analysis of crude extract expressing anti-human IgE mAb with or without SEKDEL on different dpi (2, 4, 6, 8, and 10). The protein was transferred onto a nitrocellulose membrane and the blot was probed with HRP-conjugated anti-gamma antibody. The lane number represents the harvested day post-infiltration expressing plant-produced anti-human IgE mAb. Arrowhead indicates the expression of full-length anti-human IgE mAb in plant leaves. Equal amounts of total soluble protein (7 µg) were loaded in each well. M: Protein marker; kDa: kilodalton; dpi: days post-infiltration.
