# Supplementary figures and images for: Feasibility of plant-expression system for production of recombinant anti-human IgE: An alternative production platform for therapeutic monoclonal antibodies
Source: Front Plant Sci. 2022 Dec 2;13:1012583. doi: 10.3389/fpls.2022.1012583 (PMC9755585; doi:10.3389/fpls.2022.1012583)

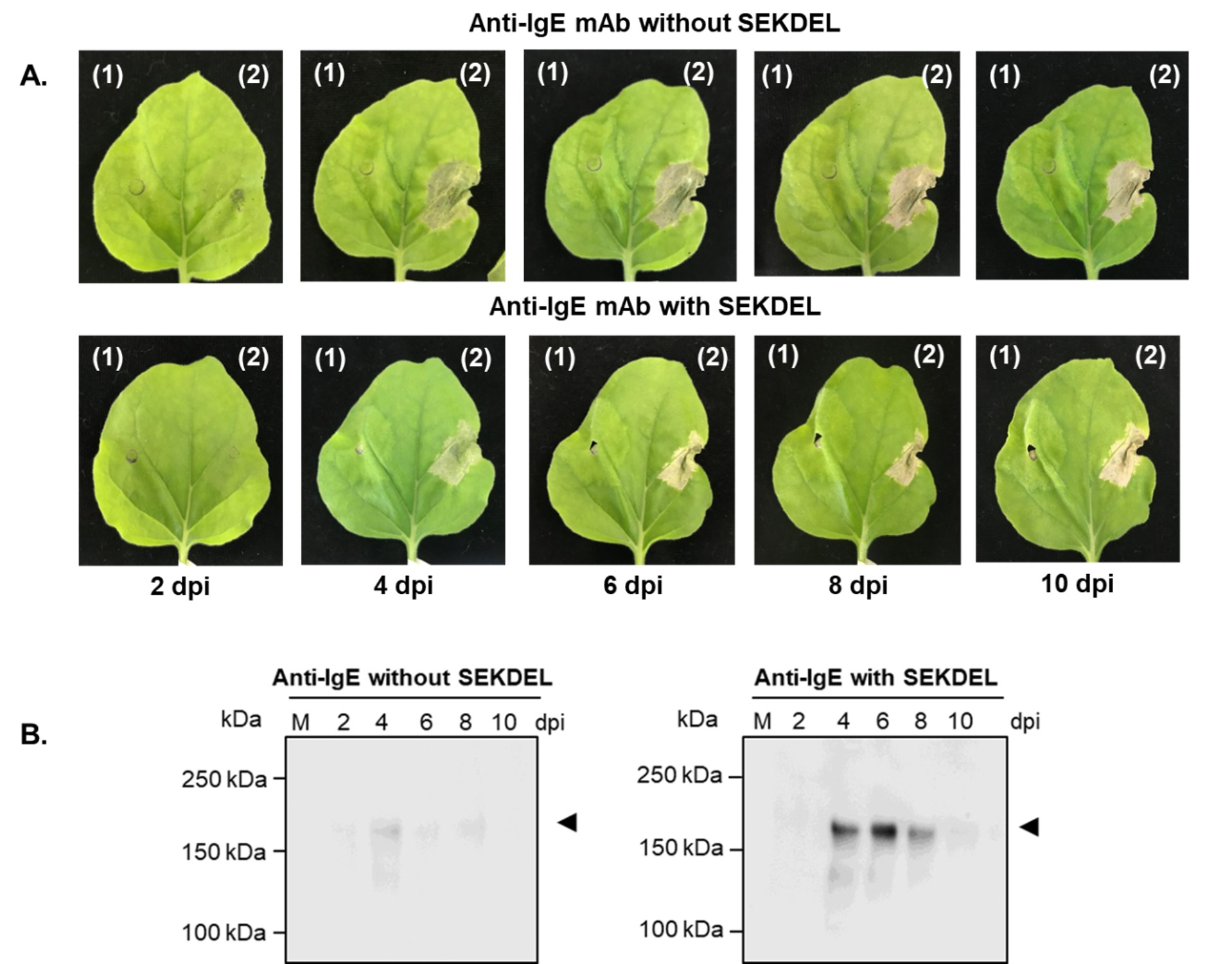

Supplement: Supplementary Figure 1 — Effect of SEKDEL signal peptide on the anti-IgE antibody expression in N. benthamiana plants. (A) Typical phenotype of N. benthamiana leaves expressing anti-human IgE mAb with or without SEKDEL on 2, 4, 6, 8, and 10 dpi; (B) Representative non-reducing western blot analysis of crude extract expressing anti-human IgE mAb with or without SEKDEL on different dpi (2, 4, 6, 8, and 10). The protein was transferred onto a nitrocellulose membrane and the blot was probed with HRP-conjugated anti-gamma antibody. The lane number represents the harvested day post-infiltration expressing plant-produced anti-human IgE mAb. Arrowhead indicates the expression of full-length anti-human IgE mAb in plant leaves. Equal amounts of total soluble protein (7 µg) were loaded in each well. M: Protein marker; kDa: kilodalton; dpi: days post-infiltration. [file Image_1.jpeg]

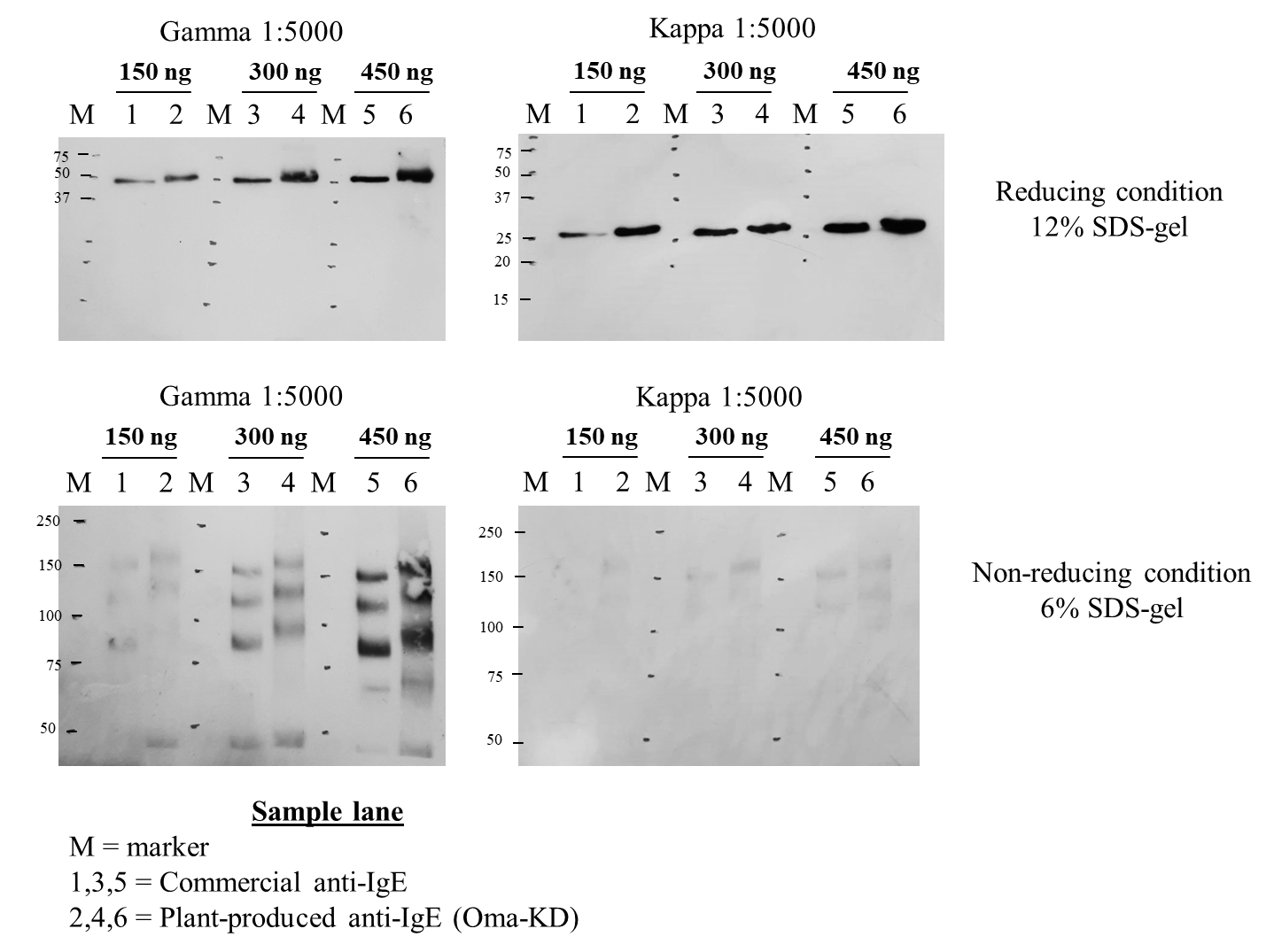


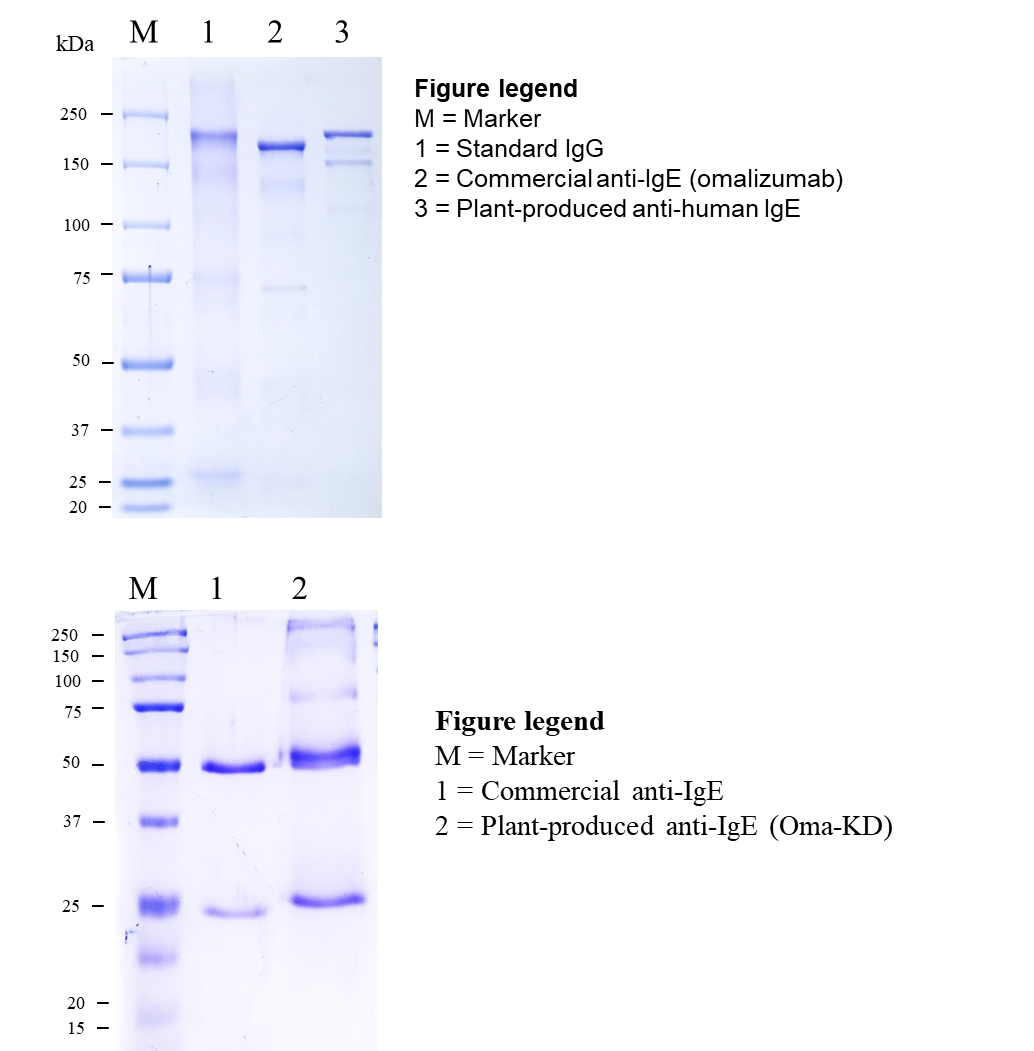


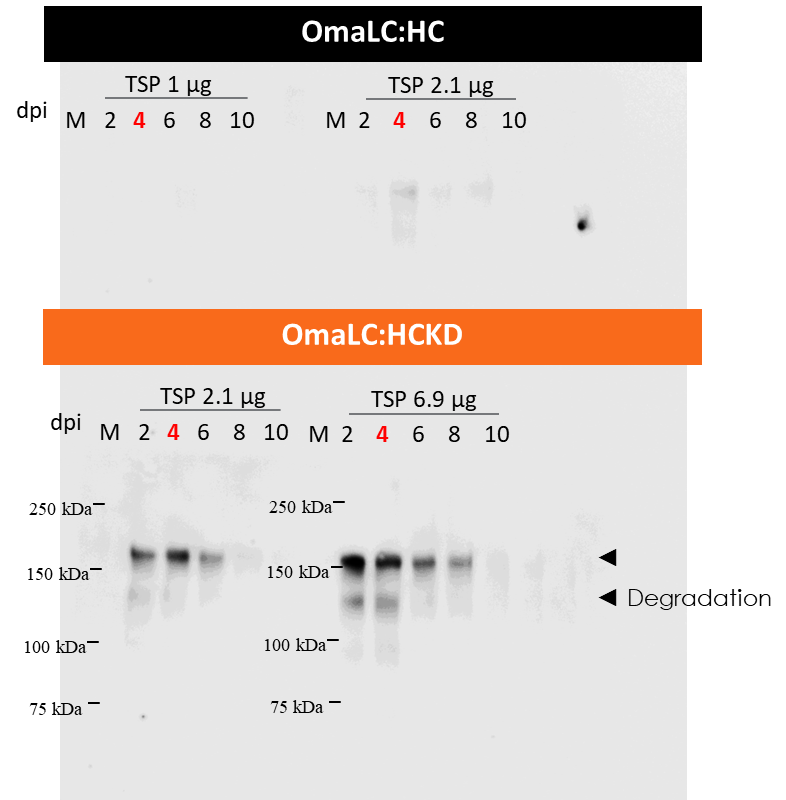

Supplement: Supplementary file 2 [file DataSheet_1.docx]
